# Point prevalence survey of antimicrobial use and healthcare-associated infections in Belgian acute care hospitals: results of the Global-PPS and ECDC-PPS 2017

**DOI:** 10.1186/s13756-019-0663-7

**Published:** 2020-01-13

**Authors:** Eline Vandael, Katrien Latour, Herman Goossens, Koen Magerman, Nico Drapier, Boudewijn Catry, Ann Versporten, the Belgian Point Prevalence Survey Study Group, Marie Andre, Samy Aouachria, Mickael Aoun, Kristof Bafort, Sofie Bartholomeus, Sophie Blumental, Anaïs Bothy, Christiane Brands, Laetitia Brassinne, Caroline Briquet, Reinoud Cartuyvels, Clara Ceyssens, Sarah Cooreman, Pierre-Yves Decleire, Philippe Declercq, Liesbeth De Cooman, Inge De Cuyper, Dirk Degraeve, Bénédicte Delaere, Mélanie Delvallée, Marjoleine Desmedt, Victoria Diaz, Ilisei Dragos, Thierry Dugernier, Elodie Elsen, Lorenzo Filippin, Eric Firre, Johan Frans, Patrick Gabriëls, Philippe Gadisseux, Michele Gerard, Bart Glibert, Truus Goegebuer, Viviane Gonissen, Antoine Harrouk, Xavier Holemans, Aline Honore, Louis Ide, Hilde Jansens, François Kidd, Annelies Koch, Deborah Konopnicki, Philippe Lefèvre, Marc Lespagnard, Sophie Lorent, Evelyne Maillart, Martine Mallet, Samuel Markowicz, Delphine Mathieu, Philippe Michel, Séverine Noirhomme, Pauline Papin, Anne Piette, Leen Pollet, Camelia Rossi, Patricia Schatt, Erica Sermijn, Astrid Sterckx, Sophia Steyaert, Walter Swinnen, Liliana Teixeira Lopes, Inge Thoelen, Myriam Turkova, Anne-Marie Van den Abeele, Valérie van den Berg, Danielle Van der beek, Vanessa Vanderper, Marc Vandevelde, Wouter Vandewal, Bruno Van Herendael, Geert Vanheule, Frederik Van Hoecke, Dana Van Kerkhoven, Ann Van Liedekerke, Lorenz Vanneste, Marc Vekemans, Christelle Vercheval, Katia Verhamme, Thessa Verniest, Veerle Westerlinck, Ingrid Wybo

**Affiliations:** 1 Healthcare-associated infections and antimicrobial resistance, Sciensano, Rue Juliette Wytsmanstraat 14, 1050 Brussels, Belgium; 2 Laboratory of Medical Microbiology, University of Antwerp, Antwerp, Belgium; 3 Belgian Antibiotic Policy Coordination Commission (BAPCOC), Direction General Healthcare, Federal Public Service Health, Food Chain Safety and Environment, Brussels, Belgium; 4 Department of Microbiology, University of Hasselt, Hasselt, Belgium; 5 Faculty of Medicine, Université Libre de Bruxelles (ULB), Brussels, Belgium

**Keywords:** Point prevalence survey, Antimicrobial consumption, Healthcare-associated infections, Belgium, Acute care hospitals

## Abstract

**Background:**

The point prevalence survey of healthcare-associated infections (HAIs) and antimicrobial use organized by the European Centre for Disease Prevention and Control (ECDC-PPS) and the Global Point Prevalence Survey of antimicrobial consumption (Global-PPS) were simultaneously performed in Belgian acute care hospitals in 2017.

**Methods:**

Belgian acute care hospitals were invited to participate in either the ECDC or Global-PPS. Hospital/ward/patient-level data were collected between September–December 2017. All patients present in the wards at 8 a.m. on the day of the PPS were included. The data of the ECDC and Global-PPS on antimicrobial consumption were pooled. Detailed data on HAIs were analysed for ECDC-PPS.

**Results:**

Overall, 110 Belgian acute care hospital sites participated in the ECDC and Global-PPS (countrywide participation rate: 81.4%, 28,007 patients). Overall, a crude prevalence of patients with at least one antimicrobial of 27.1% (95% confidence interval (CI) 26.5–27.6%) was found. The most frequently reported indications were pneumonia (23.2%), urinary tract infections (15.2%) and skin and soft tissue infections (11.9%). The reason for antimicrobial use was recorded for 81.9% of the prescriptions, a stop/review date for 40.8% and compliance with local antibiotic guidelines for 76.6%. In the ECDC-PPS, the crude prevalence of patients with at least one HAI was 7.3% (95%CI 6.8–7.7%). Most frequently reported HAIs were pneumonia (21.6%) and urinary tract infections (21.3%).

**Conclusions:**

HAI and antimicrobial use prevalence remained stable in comparison with the previous PPS (7.1% and 27.4% in 2011 and 2015, respectively). Belgian hospitals should be further stimulated to set local targets to improve antibiotic prescribing and reduce HAI.

## Introduction

Healthcare-associated infections (HAIs) and antimicrobial resistance (AMR) are well-known threats in healthcare. Point prevalence studies (PPS) have a well-established methodology to measure the prevalence of antimicrobial use and HAIs in hospitals. Results of PPS can be used to evaluate quality indicators, to follow-up antimicrobial stewardship and infection control programs, and to support decision-making [1].

In 2007, a first countrywide PPS of HAIs was conducted in 63 Belgian acute care hospitals. The prevalence of patients with at least one HAI was 6.0%. The most common infections were urinary tract infections (UTIs; 23.9%), lower respiratory tract infections (RTIs; 20.1%) and surgical site infections (SSIs; 14.6%) [2].

In 2011, the European Centre for Disease Prevention and Control (ECDC) organized a first European PPS on HAIs and antimicrobial use in European acute care hospitals. The survey’s objectives were to estimate the total burden of HAIs and antimicrobial use in acute care hospitals in the European Union (EU) and therefore provided a standardized tool for hospitals to identify targets for quality improvement [3]. The EU prevalence of patients with at least one HAI was 6.0% (country range 2.3–10.8%). In the participating Belgian acute care hospitals (*N* = 52 sites), this prevalence of patients with HAIs was 7.1% (95% confidence interval (95% CI): 6.1–8.3%). The EU prevalence of patients receiving at least one antimicrobial agent was 35.0% (country range 21.4–54.7%). In Belgium, a prevalence of antimicrobial use of 28.9% (95% CI: 26.8–31.1%) was reported [3]. In 2016–2017, a second European PPS was organized by ECDC.

Besides the ECDC-PPS, the Global Point Prevalence Survey of Antimicrobial Consumption and Resistance (Global-PPS) is organized by the University of Antwerp to monitor the ratios of antimicrobial prescribing and resistance in hospitalized inpatients on a worldwide level, with special attention to low-middle income countries. The first Global-PPS was performed in 2015. Worldwide, 34.4% of the included patients received at least one antimicrobial agent [4]. In the included Belgian acute care hospitals (*N* = 100 hospital sites), a prevalence of 27.4% was found [5]. In 2017, the second Global-PPS was set up.

The aim of this paper is to present the main results of the second ECDC-PPS and Global-PPS of antimicrobial use and HAIs in Belgian acute care hospitals in 2017.

## Methods

### Study design and participation

Two cross-sectional studies, the ECDC and Global-PPS, were simultaneously organized in Belgian acute care hospitals in 2017 by Sciensano and the Belgian Antibiotic Policy Coordination Committee (BAPCOC).

In order to collect data representative for the Belgian hospital population in the ECDC-PPS, a representative sample of hospitals was drawn using a systematic sampling design. For this, a list of all Belgian hospitals was obtained from the Federal Public Service health, Food chain safety and Environment [6]. All acute care hospitals were selected and ranked according to hospital type, total number of beds, region/province and ownership. The total number of hospitals (*N* = 102 administrative hospital groups (AHG), i.e. hospital sites that are grouped) was divided by the number to be sampled (*N* = 34) to determine the sampling interval. One substitution per hospital (runner-up) was foreseen in case of refusal of the first selected hospital.

In collaboration with Sciensano, BAPCOC invited the infection control team members and representative(s) of the antibiotic policy group within each hospital to participate in one of the two surveys. The 34 randomly selected hospitals received from Sciensano a personalized invitation and were encouraged to participate in the ECDC-PPS. Because the more complex protocol compared to the Global-PPS and higher workload, financial incentives were given to hospitals conducting the ECDC-PPS (2 euros per included patient). All inclusions in the ECDC-PPS were performed between September and November 2017. The Global-PPS was performed between September and December 2017.

### Data collection

The ECDC-PPS was organized in line with ECDC’s patient-based protocol for PPS of HAI and antimicrobial use in European acute care hospitals [7]. The protocol of the Global-PPS is available on the Global-PPS website (www.global-pps.com).

#### ECDC-PPS

Data had to be collected on one single day for each ward in the participating hospitals. The entire data collection could not exceed 2–3 weeks in a single hospital. Accident and emergency wards were excluded. A hospital form collected data on hospital type, size, beds and patients as well as a limited set of structure and process indicators (including the full-time equivalent (FTE) antimicrobial stewardship consultants, interpreted as the time that a consultant/pharmacist is specifically employed and paid for antimicrobial stewardship tasks [7]). For all patients present at the ward before or at 8 a.m. and not discharged from the ward at the time of the survey, a patient form had to be completed. This form collected patient demographic data and risk factors, use of antimicrobial agents and presence of HAIs.

Antimicrobial agents for systemic use within the Anatomical Therapeutic Chemical (ATC) groups A07AA (intestinal anti-infectives), D01BA (antifungals for systemic use), J01 (antibacterials for systemic use), J02 (antimycotics for systemic use), J04 (antimycobacterials) and P01AB (nitroimidazole-derived antiprotozoals) were included [8]. Antiviral agents (J05) and antimicrobials for the treatment of mycobacteria were excluded.

If the antimicrobial (or administration route) changed during the infection episode, the reason for change had to be registered. Possible reasons were escalation (i.e. another antimicrobial was added, the route of administration was switched from oral to parental), de-escalation (i.e. the antimicrobial was switched to a more narrow-spectrum or to a first-line antimicrobial, or other antimicrobials for the same indication were stopped), switch from intravenous to oral administration route, change because of observed or expected adverse effect of the antimicrobial and changes for another or unknown reason [7].

Infections had to be included if they met the definition of an active HAI (associated to an acute care hospital stay). An infection was considered to be active when signs and symptoms of the infection were present on the survey date or when the patient was (still) receiving treatment for that infection on the survey date. The onset of symptoms had to be on day 3 or later (day of admission = day 1) of the current admission or earlier in case the patient presenting an infection was readmitted less than 48 h after a previous admission to an acute care hospital. Exceptions to these inclusion criteria were there for SSIs, *Clostridioides difficile* infections and infections with invasive devices [7]. Microbiological test results available on the day of the PPS were collected. For a selected group of bug-drug combinations, the antimicrobial susceptibility test results (susceptible, intermediate, resistant or unknown) also had to be reported [7].

Prior to the start of the surveillance period (September 2017), several training days were organized to outline the PPS objectives and methodology to the participating hospitals. Local surveyors had to enter all data into ECDC’s HelicsWin.Net software. Thereafter, the local database had to be sent to Sciensano. All individual databases were validated, compiled and transferred to ECDC using their European Surveillance System (TESSy). The data of hospitals that participated in the ECDC-PPS were afterwards converted and imported in the Global-PPS tool (more details on this conversion in Additional file 1).

#### Global-PPS

Participating hospitals were asked to conduct the survey on one single-day and audit all in-patient wards. All patients present in the ward at 8 a.m. had to be included. Data were collected using two forms, a ward form for the recording of denominators (number of beds and number of admitted patients at 8 a.m. on the day of the PPS) and a patient form for recording detailed antimicrobial prescription (type, dose, administration route, indication, diagnosis) and resistance data for those patients who received at least one antimicrobial on the day of the PPS.

The following ATC groups were included as antimicrobial agents for systemic use: A07AA, D01BA, J01, J02, J04A, J05, P01AB and P01B (antimalarials). Additional antimicrobial quality indicators included 1) the diagnosis being documented in the patient’s notes at the start of treatment; 2) the antibiotic prescription being compliant with local guidelines and 3) if a stop or review date of the antimicrobial prescription was documented in the notes. Further, empiric or targeted treatment (based upon microbiology data from a relevant clinical specimen) was recorded. If the treatment choice was determined by available microbiology data, the participant had to indicate if it targeted a multidrug-resistant organism. Data collection forms and definitions on the different variables are available on the Global-PPS website (www.global-pps.com).

The data were entered by the participating hospitals in the freely available web-based application of the Global-PPS. This system allows anonymised data entry, validation and feedback reporting [4]. The complete database is safe-guarded at the University of Antwerp. The validated database of June 2018 was used for the analyses in this manuscript.

### Data analysis

Where possible, the data of both databases were combined and total results are shown for the Global- and ECDC-PPS (except for data that were collected only in one of the PPS). For the section on HAIs, results of each PPS were separately reported due to the differences in the methodology.

Hospital sites were classified by type (primary, secondary, tertiary or specialized) in accordance with the recommendations of ECDC and based on the list of hospitals of the Federal Public Service Health, Food chain safety and Environment [6, 7]. Total results for the Global- and ECDC-PPS were presented per ward specialty (in line with the classification of the Global-PPS); specific results for the ECDC-PPS were presented by patient specialty.

The crude prevalence of patients with at least one HAI or antimicrobial was calculated by dividing the number of patients presenting at least one HAI or antimicrobial by the total number of eligible patients. Patients presenting with multiple HAIs or prescribed multiple antimicrobials on the PPS day were thus counted only once. Prevalences were calculated along with their 95%CI.

Statistical testing was performed, using SAS Enterprise Guide statistical software, version 7.1. Means and standard deviations (SD), ranges and frequencies (%) were calculated where appropriate. The data of the ECDC and Global-PPS on antimicrobial consumption and quality indicators were pooled.

## Results

### Characteristics of the participating hospitals and included patients

Overall, 110 acute care hospital sites participated in the Global-PPS and ECDC-PPS survey in 2017. Of the random selection (*N* = 34 AHG), 16 primarily selected hospitals and 6 runner-ups participated in the ECDC-PPS. In total, 28,007 patients were included, of whom 16,207 patients in the Global-PPS and 11,800 patients in the ECDC-PPS. The characteristics of these hospital sites and their eligible patients are displayed in Tables 1 and 2, respectively.

**Table 1:** Characteristics of the included acute care hospitals in the Global and ECDC-PPS 2017 (Belgium)

Number of included	Global-PPS 2017		ECDC-PPS 2017		Total		Degree of participation^a^	
	sites	AHG^b^	sites	AHG^b^	sites	AHG^b^	sites	AHG^b^
Total	64	51	47	33	110	83	110^c^/191 = 57.6%	83/102 = 81.4%
Per type								
Primary hospitals	48	40	33	23	81	63	81/144 = 56.3%	63/77 = 81.8%
Secondary hospitals	12	7	12	8	23	14	23/27 = 85.2%	14/17 = 82.4%
Tertiary hospitals	3	3	2	2	5	5	5/9 = 55.6%	5/7 = 71.4%
Specialized hospitals	1	1	0	0	1	1	1/11 = 9.1%	1/1 = 100.0%
Per region								
Brussels	11	6	7	6	17	11	17/23 = 73.9%	11/12 = 91.7%
Flanders	33	30	16	12	49	42	49/101 = 48.5%	42/54 = 77.8%
Wallonia	20	15	24	15	44	30	44/67 = 65.7%	30/36 = 83.3%

*AHG* Administrative hospital groups, *PPS* Point prevalence survey

^a^Based on the total number of hospital sites in Belgium in 2017 (6) (total AHG: N = 102; total sites; *n* = 191)

^b^At least one site of the AHG participated

^c^One hospital participated both in the Global and ECDC-PPS 2017 (other point in time, other patients)

**Table 2:** Characteristics of the eligible patients in the Global and ECDC-PPS 2017 (Belgium, acute care hospitals)

	Global-PPS 2017		ECDC-PPS 2017		Total	
	N	%	N	%	N	%
Total number of included patients	16,207	100.0	11,800	100.0	28,007	100.0
Adults^a^	15,139	93.4	11,008	93.3	26,147	93.4
Children^a^	722	4.5	606	5.1	1328	4.7
Neonates^a^	346	2.1	186	1.6	532	1.9
Mean age ± SD^b^			60.2 ± 25.3			
Number of males / females^b^ *Missing*			5264 / 6512 *24*	44.7 / 55.2 *0.2*		
Distribution of the McCabe score (%)						
Non-fatal disease			7295	61.8		
Ultimately fatal disease			1873	15.9		
Rapidly fatal disease			689	5.8		
*Missing*			*1943*	*16.5*		
Ward specialty (%)						
Medicine	11,067	68.3	8837	74.9	19,904	71.1
Surgery	4293	26.5	2432	20.6	6725	24.0
ICU	847	5.2	531	4.5	1378	4.9
Patient specialty (%)^b^						
Medicine			3600	30.5		
Surgery			2531	21.4		
ICU			583	4.9		
Geriatrics			1813	15.4		
Obstetrics / Maternity			583	4.9		
Healthy neonates			156	1.3		
Neonatology			121	1.0		
Pediatrics			464	3.9		
Psychiatry			823	7.0		
Rehabilitation			903	7.7		
Long-term care			33	0.3		
Mix			28	0.2		
Other			50	0.4		
*Missing*			*112*	*1.0*		

*ECDC* European Centre for Disease Prevention and Control, *ICU* Intensive care unit, *N* number of patients, *PPS* point prevalence survey, *SD* Standard deviation

^a^The classification of adults, children and neonates is based on the ward type

^b^These data are only available in the ECDC-PPS whereby patient characteristics were collected for all eligible patients (as opposed to the Global-PPS where this data was only available for patients who received at least one antimicrobial)

If data were not collected in the PPS, fields were left blank.

### Antimicrobial consumption

The overarching results of the Global-PPS and ECDC-PPS will be jointly presented. Overall, the crude prevalence of patients with at least one antimicrobial was 27.1% (95%CI: 26.5–27.6%). In total, 7577 patients used one or more antimicrobials on the survey day. These patients had a mean age of 64.8 (SD: ±21.8) years and 51.3% was male. Table 3 presents the crude prevalence by ward specialty, antimicrobial subclass and hospital type. The prevalence by hospital site ranged from 2.2 to 43.3% (interquartile range: 23.0–31.4%).

**Table 3:** Crude prevalence of antimicrobial use by ward specialty/subclass/hospital type; total results for Global and ECDC-PPS 2017 (Belgium, acute care hospitals)

	Patients with at least one antimicrobial											
	N	Crude prevalence (%)	95% CI	N	Crude prevalence (%)	95% CI	N	Crude prevalence (%)	95% CI	N	Crude prevalence (%)	95% CI
Ward specialty	Total			Medicine			Surgery			ICU		
All patients	7577	27.1	26.5–27.6	4886	24.6	24.0–25.2	1988	29.6	28.5–30.7	703	51.0	48.4–53.7
Adults^a^	7095	27.1	26.6–27.7	4461	24.4	23.8–25.1	1966	29.4	28.3–30.5	668	55.1	52.3–57.9
Children^a^	430	32.4	29.9–34.9	393	31.6	29.0–34.2	22	47.8	33.4–62.3	15	38.5	23.2–53.7
Neonates^a^	52	9.8	7.3–12.3	32	7.9	5.3–10.5	0			20	15.8	9.4–22.1
Per antimicrobial subclass												
A07AA	59	0.21	0.16–0.26	52	0.26	0.19–0.33	2	0.03	0.00–0.07	5	0.36	0.05–0.68
D01BA	6	0.02	0.00–0.04	4	0.02	0.00–0.04	2	0.03	0.00–0.07	0		
J01	7323	26.2	25.6–26.7	4664	23.4	22.8–24.0	1973	29.3	28.3–30.4	686	49.8	47.1–52.4
J02	357	1.27	1.14–1.41	270	1.36	1.20–1.52	36	0.54	0.36–0.71	51	3.70	2.70–4.70
J04A	79	0.28	0.22–0.34	41	0.21	0.14–0.27	35	0.52	0.35–0.69	3	0.22	0.00–0.46
J05^b^	95	0.34	0.27–0.41	87	0.44	0.35–0.53	1	0.01	0.00–0.04	7	0.51	0.13–0.88
P01AB	149	0.53	0.45–0.62	118	0.59	0.49–0.70	27	0.40	0.25–0.55	4	0.29	0.01–0.57
Per hospital type^c^												
Primary	4866	27.0	26.3–27.6	3135	24.4	23.6–25.1	1327	30.1	28.7–31.4	404	53.1	49.5–56.6
Secondary	1768	26.1	25.0–27.1	1143	23.1	21.9–24.3	455	30.7	28.4–33.1	170	48.3	43.1–53.5
Tertiary	907	29.5	27.9–31.1	583	28.9	26.9–30.9	203	24.8	21.9–27.8	121	51.0	44.7–57.4

*CI* Confidence interval, *ECDC* European Centre for Disease Prevention and Control, *ICU* Intensive care units, *N* Total number of patients with at least one antimicrobial, *PPS* Point prevalence survey

*A07AA* intestinal antiinfectives, *D01BA* antifungals for systemic use, *J01* antibacterials for systemic use, *J02* antimycotics for systemic use, *J04A* drugs for treatment of tuberculosis, *J05* antivirals agents, *P01AB* nitroimidazole-derived antiprotozoals

^a^The classification of adults, children and neonates is based on the ward type

^b^J05 only included in the Global-PPS

^c^Results of the specialized hospital (*N* = 1) not shown

**Fig. 1:**
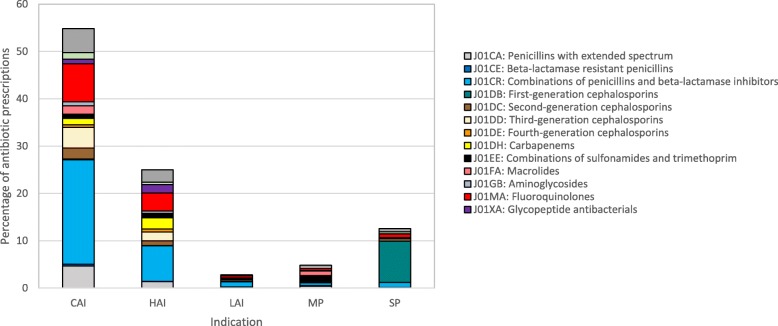
Percentage of antibiotic prescriptions per antibiotic subclass and indication, total results for Global and ECDC-PPS 2017 (Belgium, acute care hospitals). CAI = community-acquired infection, HAI = acute-hospital-acquired infection, LAI = infection acquired in long-term care facility or chronic-care hospital, MP = medical prophylaxis, SP = surgical prophylaxis. * sum of the % prescriptions CAI – HAI – LAI – MP – SP = 100%

Moreover, 1378 patients (18.2%) were treated with multiple antimicrobials on the day of the PPS. Out of all antimicrobials (*N* = 9232), 51.7% were prescribed for a community-acquired infection (CAI, *N* = 4775), 25.3% for a healthcare-associated infection (HAI, *N* = 2333), 2.7% for an infection related to a long-term care facility (LAI, *N* = 248), 5.9% for medical (*N* = 545) and 11.2% for surgical prophylaxis (*N* = 1038). Out of all antimicrobials, 8448 were antibacterials for systemic use (91.5%) for which antibiotic subclasses by indication are provided in Fig. 1. More details can be found in the supplementary data (Additional file 2). For therapeutic indications (CAI, HAI, LAI) especially ‘Penicillins in combination with a beta-lactamase inhibitor’ (J01CR; 30.1–40.2% of J01) and ‘Fluoroquinolones’ (J01MA, 14.6–16.2% of J01) were used. For medical prophylaxis, ‘Combinations of sulfonamides and trimethoprim’ (J01EE, 27.5% of J01) was the most used antibiotic subgroup. ‘First-generation cephalosporines’ (J01DB, 69.2% of J01, especially cefazolin) was most prescribed for surgical prophylaxis.

The five most frequent diagnoses for medical treatment with antimicrobials (CAI, HAI, LAI) were pneumonia (*N* = 1705, 23.2%), (lower and upper) UTIs (*N* = 1121, 15.2%), skin and soft tissue infections (*N* = 877, 11.9%), intra-abdominal sepsis (*N* = 781, 10.6%) and acute bronchitis (*N* = 522, 7.1%). Overall, the following antimicrobials were most commonly used: amoxicillin/beta-lactamase inhibitor (*N* = 1935, 21.0%), piperacillin/beta-lactamase inhibitor (*N* = 781, 8.5%), cefazolin (*N* = 726, 7.9%), ciprofloxacin (*N* = 664, 7.2%) and ceftriaxone (*N* = 350, 3.8%). Of all antimicrobial agents, 64.6% was administered parenterally (*N* = 5966) 35.2% orally (*N* = 3248), 0.13% by inhalation (*N* = 12) and 0.01% rectally (*N* = 1; missing: N = 5). More details on the diagnosis sites by indication can be found in the supplementary data (Additional file 3).

### Antimicrobial quality indicators

Antimicrobial quality indicators are presented by hospital type, ward specialty and indication in Table 4. In general, the reason for antimicrobial use was recorded for 81.9% of the prescriptions and a stop/review date was known for 40.8% of the prescriptions. For antibiotic prescriptions, a compliance with local antibiotic guidelines was reported in 76.6%. For surgical prophylaxis, this compliance was 73.2%. The duration of surgical prophylaxis was in 35.1% of the cases a single dose, in 39.7% 1 day (multiple doses) and in 25.2% more than 1 day. For prolonged surgical prophylaxis (> 1 day), prophylaxis for plastic or orthopedic surgery (30.8%) and prophylaxis for urological surgery (27.7%) were most often registered as diagnosis (only registered in the Global-PPS).

**Table 4:** Overview of the antimicrobial quality indicators, total results for Global and ECDC-PPS 2017 (Belgium, acute care hospitals)

	Targeted treatment		Targeted treatment (resistant MO)		Reason recorded		Stop or review date recorded		Parenteral administration		Guidelines available		Compliant to local guidelines	
	N	%	N	%	N	%	N	%	N	%	N	%	N	%
All hospital sites	1242	37.0	226	6.7	7558	81.9	2093	40.8	5072	69.3	3686	88.4	2825	76.6
Per type of hospital^a^														
Primary	804	33.9	156	6.6	4754	83.2	1362	39.8	3280	69.3	2508	86.1	1935	77.2
Secondary	248	44.4	31	5.6	1860	84.8	480	53.0	1162	68.1	689	95.8	569	82.6
Tertiary	185	45.7	37	9.1	889	70.1	237	32.0	606	71.4	455	89.9	289	63.5
Ward specialty														
Medicine	821	34.6	125	5.3	5007	84.5	1282	39.3	2911	62.4	2353	88.9	1820	77.3
Surgery	279	43.3	60	9.3	1726	73.3	600	45.8	1501	76.1	969	87.2	703	72.5
ICU	142	42.0	41	12.1	825	86.4	211	38.2	660	96.2	364	88.1	302	83.0
Indication														
CAI	684	30.7	89	4.0	4308	90.2	1001	37.5	2647	68.0	2021	90.3	1626	80.5
HAI	523	51.1	129	12.6	2011	86.2	551	41.1	1250	71.4	911	86.7	713	78.3
LAI	35	34.7	8	7.9	231	93.1	64	53.8	137	68.2	92	89.3	76	82.6
MP	NA	NA	NA	NA	305	56.0	56	19.4	93	28.4	131	74.4	88	67.2
SP	NA	NA	NA	NA	640	61.7	377	82.3	859	89.5	414	91.6	303	73.2

*ECDC* European Centre for Disease Prevention and Control, *CAI* Community-acquired infections, *HAI* Healthcare-associated infections, *ICU* Intensive care unit, *LAI* Infection present on admission from long-term care facility or Nursing Home, *MO* Micro-organisms, *MP* Medical prophylaxis, *N* Number of prescriptions or patients, *NA* Not applicable, *PPS* Point prevalence survey, *SP* Surgical prophylaxis

^a^Results of the specialized hospital (*N* = 1) not shown

**Targeted treatment:** only patients included in the Global-PPS and with at least one antibiotic medical (CAI, HAI, LAI) treatment (*N* = 3354)

**Reason recorded:** patients of the Global- and ECDC-PPS; all antimicrobial prescriptions included

**Stop or review date recorded:** only patients included in the Global-PPS; all antimicrobial prescriptions included

**Parenteral administration:** patients of the Global- and ECDC-PPS; patients who received at least one parenteral antibiotic for systemic use over all patients who received at least one antibiotic for systemic use

**Guidelines available:** only patients included in the Global-PPS; antibiotic prescriptions for which guidelines were available, count at patient level and diagnosis (for combination therapy: no guidelines available for ≥1 antibiotic = no guideline for combination)

**Compliant to local guidelines:** only patients included in the Global-PPS; antibiotic prescriptions for which guidelines were available used as denominator, count at patient level and diagnosis (for combination therapy no compliance for ≥1 antibiotic = combination not compliant)

In the Global-PPS, 37.9% of therapeutic antimicrobial prescribing (CAI, HAI, LAI) was based on a microbiological result (targeted treatment (*N* = 1566), whereby following resistant micro-organisms were most often reported: extended-spectrum beta-lactamase (ESBL) producing *Enterobacteriaceae* (*N* = 76 patients), third generation cephalosporin resistant *Enterobacteriaceae* (non-ESBL producing or ESBL status unknown) (*N* = 39 patients) and methicillin-resistant *Staphylococcus aureus* (MRSA) (*N* = 26 patients).

In the ECDC-PPS, a change in the antimicrobial treatment was registered for 24.7% of the antimicrobials (*N* = 1012): escalation in 425 cases (10.4%), switch from intravenous to oral in 256 cases (6.2%), de-escalation in 237 cases (5.8%), change due to an adverse effect in 21 cases (0.5%), and change for another of unknown reason in 73 cases (1.8%).

The median number of FTEs for antimicrobial stewardship consultants, registered in hospitals participating in the ECDC-PPS (*N* = 29), was 0.29 per 250 beds (interquartile range 0.20–0.55).

### Healthcare-associated infections

#### ECDC-PPS 2017

In the subset of 47 hospital sites (11,800 included patients) that participated in the ECDC-PPS 2017, the crude prevalence of patients with at least one HAI was 7.3% (95%CI 6.8–7.7%, range per hospital site: 0.0–18.1%). The crude prevalences are shown by hospital type and patient specialty in Table 5. For 5.6% (48/856) of the patients with at least one HAI, no antimicrobial medical treatment (prophylaxis excluded) was registered. Based on the indication of antimicrobial prescriptions, the prevalence of patients with a treatment for a HAI was 6.8% (95%CI 6.3–7.2%).

**Table 5:** Crude prevalence of HAI and antimicrobial use, ECDC-PPS 2017 (Belgium, acute care hospitals)

	Total number of patients	Patients with at least one HAI		
		N	Crude prevalence (%)	95% CI
Total prevalence	11,800	856	7.3	6.8–7.7
Total prevalence with exclusion of psychiatry, rehabilitation and long-term care	10,041	776	7.7	7.2–8.3
Prevalence by hospital type				
Primary	7214	489	6.8	6.2–7.4
Secondary	3337	253	7.6	6.7–8.5
Tertiary	1249	114	9.1	7.5–10.7
Prevalence by patient specialty				
Medicine	3600	265	7.4	6.5–8.2
Surgery	2531	204	8.1	7.0–9.1
Intensive care	583	122	20.9	17.6–24.2
Geriatrics	1813	158	8.7	7.4–10.0
Obstetrics/ Maternity	583	9	1.5	0.5–2.5
Healthy neonates	156	0	0.0	
Neonatology	121	4	3.3	0.1–6.6
Pediatrics	464	12	2.6	1.1–4.0
Psychiatry	823	9	1.1	0.4–1.8
Rehabilitation	903	67	7.4	5.7–9.1
Long-term care	33	4	12.1	1.0–23.3
Mix	28	0	0.0	
Other	50	2	4.0	0.0–9.4

*CI* Confidence interval, *ECDC* European Centre for Disease Prevention and Control, *HAI* Healthcare-associated infection, *N* Number of patients with at least one HAI/antimicrobial, *PPS* Point prevalence survey

In total, 911 HAIs were registered of which 17.9% (*N* = 163) were already present on hospital admission. In most cases, the HAI was linked to the current hospital (*N* = 810, 88.9%) and with the current ward (*N* = 617, 67.7%). In 20.9% (*N* = 190) of the cases, the HAI was linked to an invasive device. In Table 6, the main groups of HAIs are displayed by patient specialty. The most common HAIs were pneumonia (*N* = 197, 21.6%) and UTIs (*N* = 194, 21.3%).

**Table 6:** Distribution of main groups of HAIs by patient specialty, ECDC-PPS 2017 (Belgium, acute care hospitals)

Patient Specialty	Number (%) of infections by main HAI group															Total (%)
	BJ	BSI	CNS	CRI	CVS	EENT	GI	LRTI	NEO	PN	REPR	SSI	SST	SYS	UTI	
Medicine	2 (0.7)	40 (14.2)		7 (2.5)	3 (1.1)	10 (3.6)	32 (11.4)	10 (3.6)		69 (24.6)	1 (0.4)	16 (5.7)	14 (5.0)	20 (7.1)	57 (20.3)	281 (30.8)
Surgery	3 (1.4)	21 (9.6)	3 (1.4)	3 (1.4)	2 (0.9)	1 (0.5)	15 (6.9)	2 (0.9)		20 (9.1)	1 (0.5)	95 (43.4)	8 (3.7)	6 (2.7)	39 (17.8)	219 (24.0)
Intensive care		24 (17.8)	1 (0.7)	2 (1.5)	3 (2.2)		9 (6.7)	14 (10.4)		49 (36.3)		15 (11.1)	1 (0.7)	5 (3.7)	12 (8.9)	135 (14.8)
Geriatrics		15 (9.0%)		2 (1.2)		3 (1.8)	22 (13.3)	10 (6.0)		43 (25.9)		5 (3.0)	7 (4.2)	5 (3.0)	54 (32.5)	166 (18.2)
Obstetrics/Maternity										1 (10.0)		5 (50.0)	1 (10.0)		3 (30.0)	10 (1.1)
Healthy neonates																0
Neonatology		1 (25.0)						1 (25.0)		1 (25.0)				1 (25.0)		4 (0.4)
Pediatrics		1 (8.3)				2 (16.7)	3 (25.0)	1 (8.3)		1 (8.3)		2 (16.7)		1 (8.3)	1 (8.3)	12 (1.3)
Psychiatry								2 (22.2)		1 (11.1)			2 (22.2)		4 (44.4)	9 (1.0)
Rehabilitation	1 (1.5)	2 (2.9)			1 (1.5)	2 (2.9)	5 (7.4)	5 (7.4)		11 (16.2)		14 (20.6)	1 (1.5)	2 (2.9)	24 (35.3)	68 (7.5)
Long-term care		1 (20.0)								1 (20.0)		2 (40.0)	1 (20.0)			5 (0.5)
Mix																0
Other						1 (50.0)	1 (50.0)									2 (0.2)
Total	6 (0.7)	105 (11.5)	4 (0.4)	14 (1.5)	9 (1.0)	19 (2.1)	87 (9.6)	45 (4.9)	0	197 (21.6)	2 (0.2)	154 (16.9)	35 (3.8)	40 (4.4)	194 (21.3)	911

*BJ* Bone and joint infection, *BSI* Bloodstream infection, *CNS* Central nervous system infection, *CRI* Catheter-related infection, *CVS* Cardiovascular infection, *ECDC* European Centre for Disease Prevention and Control, *EENT* Eye, ear, nose or mouth infection, *GI* Gastro-intestinal infection, *HAI* Healthcare-associated infection, *LRTI* Lower respiratory tract infection, *N* Number of infections, *NEO* Specific neonatal cases, *PN* Pneumonia, *PPS* Point prevalence survey, *REPR* Reproductive tract infection, *SSI* Surgical site infection, *SST* Skin and soft tissue infection, *SYS* Systemic infection, *UTI* Urinary tract infection

A positive microbiological result was reported for 62.0% of the HAIs (*N* = 565/911). In total, 721 microorganisms were documented. The most commonly isolated micro-organisms were *Escherichia coli* (*N* = 162, 17.8%), *Staphylococcus aureus* (N = 81, 8.9%) and *Pseudomonas aeruginosa* (N = 47, 5.2%). In the susceptibility tests, a resistant result was reported for 97 tests (susceptible: *N* = 793, intermediate: N = 3, not available or unknown: *N* = 454). More details on the intermediate and resistant micro-organisms are presented in Additional file 4.

#### Global-PPS 2017

In the Global-PPS 2017 (64 hospital sites, 16,207 included patients), the prevalence of patients with at least one HAI (based on the indication of antimicrobial prescriptions) was 6.8% (95%CI 6.4–7.2%, hospital site range: 2.0–12.1%). Similar as in the ECDC-PPS, the HAI prevalence was highest in tertiary hospitals (10.6% [95%CI 9.2–12.0%]) and in ICU (22.0% [95%CI 19.2–24.8%]).

## Discussion

### Main results and comparison with previous PPS in Belgian acute care hospitals

#### Prevalence of antimicrobial consumption

A crude prevalence of patients with at least one antimicrobial of 27.1% was found. This prevalence is slightly lower than the Belgian results in the previous ECDC-PPS (28.9%) and Global-PPS (27.4%) conducted in 2011 and 2015, respectively. The proportion of medical (5.9%) and surgical prophylaxis (11.2%) decreased in comparison with 2011 (9.0% and 11.8% respectively). The top 3 of most used antimicrobial agents (amoxicillin/beta-lactamase inhibitor, piperacillin/beta-lactamase inhibitor, cefazolin) and the percentage of antimicrobials administered parenterally (64.6%) remained the same as in 2011 and 2015 [3, 5]. In the participating European hospitals in the ECDC-PPS 2016–2017, a higher percentage of antimicrobials were administered parenterally (72.8%) [9]. These results also correspond with the results of the national surveillance of antimicrobial consumption in Belgian hospitals (BeH-SAC) [10, 11].

#### Antimicrobial quality indicators

BAPCOC published an action plan 2014–2019 with specific targets for the hospital setting that should be accomplished by 2019 [12]. The first target is that 90% of antibiotic prescriptions should be in line with the local guidelines. In the current study, a compliance of 76.6% was reported. This compliance was lowest in tertiary hospitals (63.5%, but the highest proportion of targeted treatment: 45.7%) and for medical prophylaxis (67.2%). Only in 88.4% of the prescriptions it was indicated that a local guideline was available. In the Global-PPS 2015, guideline availability (90.8%) and compliance (79.5%) was slightly higher [5].

Specifically for surgical prophylaxis, 90% compliance with local guidelines for both the choice and duration of treatment is targeted by BAPCOC [12]. In 2017, the compliance concerning the choice of drug was 73.2%. First-generation cephalosporins (especially cefazolin) were prescribed in 69.2%, followed by penicillins in combination with a beta-lactamase inhibitor (8.8%) and fluoroquinolones (5.9%). The duration of surgical prophylaxis was prolonged (> 1 day) in 25.2%. In 2015, the mean proportion of prolonged surgical prophylaxis cases was 28.2% and cefazolin was prescribed in 62.6% [5]. Penicillins with a beta-lactamase inhibitor and fluoroquinolones are still prescribed in a considerable amount of cases. BAPCOC performed audits on surgical prophylaxis in hospitals in 2013 and 2017; choice and duration compliance are expected to further improve. More detailed analyses are needed to allow target setting for specific indications.

The action plan of BAPCOC stated that the indication of the prescription should be available in the medical file in 90% [12]. This study shows that this target is not yet achieved (reason for antimicrobial use was recorded in 81.9%). Finally, post-prescription review is also an important antimicrobial quality indicator to prevent unnecessary prolonged antimicrobial use [13]. The Global-PPS showed the availability of a stop/review date in only 40.8% of the prescriptions.

#### Prevalence of healthcare-associated infections

Based on the ECDC-PPS 2017 data, a crude prevalence of patients with at least one HAI of 7.3% was detected in Belgian acute care hospitals. The highest prevalences were found in tertiary hospitals (9.1%) and on ICU (20.9%). In comparison with the ECDC-PPS in 2011, this prevalence remained stable (7.1%) [3]. The most frequently reported HAIs were pneumonia, UTIs and SSIs, which is in line with the previous PPS [3].

### Comparison with other European countries

The overall prevalence of antimicrobial consumption in Belgian acute care hospitals (27.1%) is lower than reported for the participating European hospitals in the ECDC-PPS 2016–2017 (weighted prevalence: 30.3% [95%CI 29.0–31.6%]). In addition, the amount of prolonged surgical prophylaxis (> 1 day) in Belgian acute care hospitals (25.2%) was clearly lower than in the participating European hospitals (54.2%, country range 19.8–95.0%) [9]. Belgian acute care hospitals reported a higher number of FTE for antimicrobial stewardship per 250 beds (Belgian median 0.29 versus the European median 0.08, country range 0–0.60) [9]. In contrary, the prevalence of patients with at least one HAI in Belgian acute care hospitals (7.3%) remains clearly higher than in other European hospitals. A weighted prevalence of HAI of 5.5% (95%CI 4.5–6.6%) was reported in the participating European hospitals in the ECDC-PPS 2016–2017 [14]. In Scottish hospitals which performed the ECDC-PPS in 2016, a prevalence of antimicrobial use of 35.7% (95%CI 34.2–37.2%) and HAI prevalence of 4.6% (95%CI 4.1–5.1%) were detected in acute adult patients [15].

### Strengths and limitations

In 2017, two PPS were performed in Belgian acute care hospitals in line with the international standardized methodology of ECDC-PPS and Global-PPS. The added value of combining the data of the two surveys is that it resulted in a large database with a participation rate of more than 80% of all Belgian acute care hospitals. Moreover, each PPS system has its own added value (e.g. ECDC-PPS: more detailed data on HAIs, Global-PPS: other quality indicators), both at local and national level, resulting in a more complete picture on the current practice in Belgian acute care hospitals and allowing international comparisons. To our knowledge, it is the first time that data of both PPS using a similar methodology are combined. All participating hospitals already received an individual feedback of their prevalence data, benchmarked with national results. Participation, if organised countrywide, is also recorded as a mandatory quality indicator to improve infection control in Belgian hospitals [16].

Some limitations should be acknowledged. As this was a cross-sectional survey, only prevalences can be reported and patients were not followed-up in time. Prevalence study only show a snapshot of the situation, therefore they are not ideal for measuring for example AMR. However, in addition to the PPS, different (mandatory and voluntary) surveillances on AMR and HAIs are conducted in Belgian hospitals (e.g. surveillances on blood-stream infections, *Clostridioides difficile*, SSI, MRSA, vancomycin resistant enterococci (VRE), multi-resistant Gram negative bacteria) [10]. Secondly, the results were not corrected for patient case mix or institutional factors. Therefore, comparison with the results of different PPS should be interpreted carefully as different hospitals participated each time. Detected differences between several PPS might be explained by differences in case mix. In addition, the definition of the type of hospitals might have been different in past PPS. Finally, although we managed to merge the data of both PPS in the best way (see Additional file 1), some differences in the protocols of the ECDC-PPS and Global-PPS could have had a small influence on the results (e.g. antivirals (J05, 95 prescriptions: 1.03%) and antimalarials (P01B, 3 prescriptions: 0.03%) were only included in the Global-PPS). As only one hospital participated in both surveys, a direct comparison of the results obtained from both study methods was not assessed here.

### Future perspectives

It is recommended that PPS are repeatedly performed to follow-up the evolution over time and monitor the impact of antimicrobial stewardship and infection prevention programmes [1]. The Global-PPS tool is continuously available and hospitals themselves can decide how often they conduct a PPS. ECDC aims to organize a PPS every 5 years (third ECDC-PPS probably planned in 2022). On a national level, a fixed time-interval (e.g. every 2 years) should be defined to be able to provide data for benchmarking and to evaluate targets for example as set up in the 2014–2019 action plan of BAPCOC. Since 2018, Belgian hospitals are partly financed based on the level of quality in their hospital (Pay for Performance project), and participation in a PPS is one of the conditions for the financing [17]. There is still a large range in the prevalence of antimicrobial consumption (2.2 to 67.3%) and of HAIs (0.0 to 18.1%) between hospital sites. Hospitals with outlying results should further be targeted to help the local antibiotic policy teams develop specific antimicrobial stewardship and infection prevention programmes to improve.

In general, concerning the antimicrobial consumption, the high prescribing of ‘Fluoroquinolones’ (J01MA) is a concern and should be a target for intervention. In addition, the results of the discussed antimicrobial quality indicators should be further improved to reach the BAPCOC targets (90% for all indicators). Moreover, the number of patients who develop a HAI in Belgian acute care hospitals remains high. The reasons for these high rates of HAIs should be further investigated.

## Conclusions

In comparison with previous PPS, the prevalence of antimicrobial use and HAI and most results for the antimicrobial quality indicators remained status quo. Belgian hospitals should be further stimulated to regularly participate in a PPS and to set local targets for improving antibiotic prescribing and reducing HAI.

## Supplementary information

**Additional file 1:** **Table S1.** Overview how the ECDC-PPS data were converted in the Global-PPS database (Belgium, acute care hospitals, 2017).

**Additional file 2:** **Table S2.** Overview of antibiotic (J01) prescriptions by antibiotic subclass (ATC level 4) and by indication, total results for Global and ECDC-PPS 2017 (Belgium, acute care hospitals).

**Additional file 3:** **Table S3.** Description of diagnosis sites of the antimicrobial prescriptions per indication, total results for Global and ECDC-PPS 2017 (Belgium, acute care hospitals).

**Additional file 4:** **Table S4.** Overview of the number of isolates (selected bug-drug combinations) with known antimicrobial susceptibility testing results (AST; first-level antimicrobial resistance (AMR) markers combined) for healthcare-associated infections (HAIs) and resistant results to the antimicrobials included in the protocol, ECDC-PPS 2017 (Belgium, acute care hospitals).

## Data Availability

The datasets used and/or analysed during the current study are available from the corresponding author on reasonable request.

## Abbreviations

AHG: Administrative hospital groups
AMR: Antimicrobial resistance
ATC: Anatomical Therapeutic Chemical
BAPCOC: Belgian Antibiotic Policy Coordination Committee
BJ: Bone and joint infection
BSI: Bloodstream infection
CAI: Community-acquired infections
CI: Confidence interval
CNS: Central nervous system infection
CRI: Catheter-related infection
CVS: Cardiovascular infection
ECDC: European Centre for Disease Prevention and Control
EENT: Eye, ear, nose or mouth infection
ESBL: Extended-spectrum beta-lactamase
EU: European Union
FTE: Full-time equivalent
GI: Gastro-intestinal infection
HAI: Healthcare-associated infection
ICU: Intensive care unit
LAI: Infection present on admission from long-term care facility or Nursing Home
LRTI: Lower respiratory tract infection
MO: Micro-organisms
MP: Medical prophylaxis
MRSA: Methicillin-resistant *Staphylococcus aureus*
N: Number
NA: Not applicable
NEO: Specific neonatal cases
PN: Pneumonia
PPS: Point prevalence survey
REPR: Reproductive tract infection;
RTI: Respiratory tract infection
SD: Standard deviation
SP: Surgical prophylaxis
SSI: Surgical site infection
SST: Skin and soft tissue infection
SYS: Systemic infection
UTI: Urinary tract infection
VRE: Vancomycin resistant enterococci
