# Supplementary material for: Point prevalence survey of antimicrobial use and healthcare-associated infections in Belgian acute care hospitals: results of the Global-PPS and ECDC-PPS 2017
Source: Antimicrob Resist Infect Control. 2020 Jan 13;9:13. doi: 10.1186/s13756-019-0663-7 (PMC6958935; doi:10.1186/s13756-019-0663-7)
Supplement: Supplementary file 1 — Additional file 1: Table S1. Overview how the ECDC-PPS data were converted in the Global-PPS database (Belgium, acute care hospitals, 2017). [file 13756_2019_663_MOESM1_ESM.pdf]

Point Prevalence Survey of Antimicrobial Use and Healthcare-Associated Infections in Belgian Acute Care Hospitals: Results of the Global-PPS and ECDC-PPS 2017

ADDITIONAL FILE 1

Table S1: Overview how the ECDC-PPS data were converted in the Global-PPS database (Belgium, acute care hospitals, 2017)

Variables	ECDC-PPS	Global-PPS	Comments
**Department forms**			
Survey date	DateOfSurveyWard	Survey date	
Department Name	UnitIdWard	Department Name	
Department Description	UnitSpecialtyWard	Department Description	
Total admitted patients	Number of included patients per ward	Total admitted patients	
Total available beds	NumWardBeds	Total available beds	
Department Type	SUR	Adult Surgical Ward (ASW)	Manually checked per hospital based on the description of the ward (based on UnitSpecialtyWard + Full ward name)
	GER	Adult Medical Ward (AMW)	
	MED	Adult Medical ward / Haematology-Oncology MW	
	MIX	Based on patient speciality, highest number	
	O	Adult Medical ward / Haematology-Oncology MW	
	ICU	Adult ICU of PICU of NICU	
	PED	Paediatric MW of SW of H-O of transplant	
	RHB	Adult Medical Ward (AMW)	
	GO	Adult Medical Ward (AMW)	
	PSY	Adult Medical Ward (AMW)	
	NEO	Neonatal MW	
	LTC	Adult Medical Ward (AMW)	
Ward specialty (activity code)		Adult ICU = ICU	Based on the department type
		Adult Medical Ward (AMW) = M	
		Adult Surgical Ward (ASW) = S	
		Adult Haematology-Oncology MW = M	

		Paediatric Haematology-Oncology MW = M	
		NICU = ICU	
		Neonatal MW = M	
		PICU = ICU	
		Paediatric MW = M	
		Paediatric SW = S	
		Pneumology AMW = M	
		Adult Transplant (BMT/solid) MW = M	
		Paediatric Transplant (BMT/Solid) MW = M	
Mixed department	*Missing*	Mixed department	Not coded for ECDC-PPS data
**Patients forms**			
Department Name	UnitId	Department Name	
Pat-ID (anonym)	RecordId	Pat-ID (anonym)	
Age years	Age	Age years	
Age months	AgeMonth	Age months	
Weight	*Missing*	Weight	Not coded for ECDC-PPS data
Gender	Gender	Gender	
Treatment based on biomarker data	*Missing*	Treatment based on biomarker data	Not coded for ECDC-PPS data
Biomarker name	*Missing*	Biomarker name	Not coded for ECDC-PPS data
Biomarker fluid	*Missing*	Biomarker fluid	Not coded for ECDC-PPS data
Biomarker value	*Missing*	Biomarker value	Not coded for ECDC-PPS data
Biomarker unit	*Missing*	Biomarker unit	Not coded for ECDC-PPS data
ATC code	ATCCode	ATC code	
Single Unit Dose	DoseStrength	Single Unit Dose	
Unit	DoseStrengthUnit	Unit	
Doses/day	NumberOfDoses	Doses/day	
Route	AntimicrobialRoute	Route	
	BAC/ASB	BAC	

Diagnosis code	BJ-O/BJ-SSI	BJ	Based on Antimicrobial Diagnosis in the ECDC-PPS, only available in case of treatment, not registered for prophylaxis (=missing)
	BRON	Bron	
	CNS	CNS	
	CVS	CVS	
	CYS	Cys	
	ENT	ENT	
	Missing	EYE	
	FN	FN	
	GI	GI	
	GUM	GUM	
	Missing	HIV	
	IA	IA	
	Missing	LUNG	
	Missing	LYMPH	
	Missing	Malaria	
	Missing	MP-GEN	
	Missing	MP-MAT	
	Missing	NEO-MP	
	OBGY	OBGY	
	CF	Other	
	PNEU	Pneu	
	Missing	PROK	
	Missing	Proph BJ	
	Missing	Proph CNS	
	Missing	Proph CVS	
	Missing	Proph ENT	
	Missing	Proph EYE	
	Missing	Proph GI	
	Missing	Proph OBGY	

	Missing	Proph RESP	
	Missing	Proph UTI	
	Missing	PUO	
	Missing	PUO-HO	
	PYE	Pye	
	CSEP/SIRS	SEPSIS	
	SST-O/SST-SSI	SST	
	Missing	TB	
	NA/UNK/UND	UNK	
	Missing	URTI	
Indication	CI	CAI	Based on Antimicrobial Indication, not possible to further specify for HAI so all classified as ‘other’ (except for LI)
	HI	HAI1	
		HAI2	
		HAI3	
		HAI4	
		HAI5	
	LI	HAI6	
	MP	MP	
	O	OTH	
	SP1	SP1	
	SP2	SP2	
	SP3	SP3	
	UNK/UI	UNK	
Reason in notes	ReasonInNotes	Reason in notes	
Guideline Compliance	*Missing*	Guideline Compliance	Not coded for ECDC-PPS data
Is a stop/review date documented	*Missing*	Is a stop/review date documented	Not coded for ECDC-PPS data
Targeted treatment	*Missing*	Treatment	Not coded for ECDC-PPS data

MRSA	*Missing*	MRSA	Not coded for ECDC-PPS data
MRCoNS	*Missing*	MRCoNS	Not coded for ECDC-PPS data
VRE	*Missing*	VRE	Not coded for ECDC-PPS data
ESBL-producing Enterobacteriaceae	*Missing*	ESBL-producing Enterobacteriaceae	Not coded for ECDC-PPS data
3rd generation cephalosporin resistant Enterobacteriaceae non-ESBL producing or ESBL status unknown	*Missing*	3rd generation cephalosporin resistant Enterobacteriaceae non-ESBL producing or ESBL status unknown	Not coded for ECDC-PPS data
Carbapenem-resistant Enterobacteriaceae	*Missing*	Carbapenem-resistant Enterobacteriaceae	Not coded for ECDC-PPS data
ESBL-producing non fermenter Gram-negative bacilli	*Missing*	ESBL-producing non fermenter Gram-negative bacilli	Not coded for ECDC-PPS data
Carbapenem-resistant non fermenter Gram-negative bacilli	*Missing*	Carbapenem-resistant non fermenter Gram-negative bacilli	Not coded for ECDC-PPS data
Targeted treatment against other MDR organisms	*Missing*	Targeted treatment against other MDR organisms	Not coded for ECDC-PPS data

Abbreviations are explained in the ECDC-PPS 2016-2017 protocol (7) and on the website of the Global-PPS (www.global-pps.com
