# Supplementary material for: Point prevalence survey of antimicrobial use and healthcare-associated infections in Belgian acute care hospitals: results of the Global-PPS and ECDC-PPS 2017
Source: Antimicrob Resist Infect Control. 2020 Jan 13;9:13. doi: 10.1186/s13756-019-0663-7 (PMC6958935; doi:10.1186/s13756-019-0663-7)
Supplement: Supplementary file 2 — Additional file 2: Table S2. Overview of antibiotic (J01) prescriptions by antibiotic subclass (ATC level 4) and by indication, total results for Global and ECDC-PPS 2017 (Belgium, acute care hospitals). [file 13756_2019_663_MOESM2_ESM.pdf]

**Point Prevalence Survey of Antimicrobial Use and Healthcare-Associated Infections in Belgian Acute Care Hospitals: Results of the Global-PPS and ECDC-PPS 2017**

ADDITIONAL FILE 2

**Table S2: Overview of antibiotic (J01) prescriptions by antibiotic subclass (ATC level 4) and by indication, total results for Global and ECDC-PPS 2017 (Belgium, acute care hospitals)**

| ATC-code (level 4) | Name | Number of prescriptions (% J01) | | | | | |
|---|---|---|---|---|---|---|---|
| | | Total | CAI | HAI | LAI | MP | SP |
| **J01** | **Antibacterials for systemic use** | **8448** | **4511** | **2057** | **229** | **396** | **1033** |
| J01AA | Tetracyclines | 55 (0.7%) | 30 (0.7%) | 19 (0.9%) | 2 (0.9%) | 1 (0.3%) | 0 |
| J01BA | Amphenicols | 3 (0.04%) | 1 (0.02%) | 0 | 0 | 0 | 0 |
| J01CA | Penicillins with extended spectrum | 562 (6.7%) | 382 (8.5%) | 111 (5.4%) | 19 (8.3%) | 32 (8.1%) | 6 (0.6%) |
| J01CE | Beta-lactamase sensitive penicillins | 49 (0.6%) | 33 (0.7%) | 4 (0.2%) | 0 | 11 (2.8%) | 1 (1.0%) |
| J01CF | Beta-lactamase resistant penicillins | 233 (2.8%) | 133 (2.9%) | 89 (4.3%) | 1 (0.4%) | 1 (0.3%) | 4 (0.4%) |
| J01CG | Beta-lactamase inhibitors | 7 (0.08%) | 4 (0.9%) | 3 (0.1%) | 0 | 0 | 0 |
| J01CR | Combinations of penicillins, incl. beta-lactamase inhibitors | 2724 (32.3%) | 1814 (40.2%) | 619 (30.1%) | 91 (39.7%) | 49 (12.4%) | 91 (8.8%) |
| J01DB | First-generation cephalosporins | 737 (8.7%) | 13 (0.3%) | 4 (0.2%) | 1 (0.4%) | 3 (0.8%) | 715 (69.2%) |
| J01DC | Second-generation cephalosporins | 347 (4.1%) | 193 (4.3%) | 82 (4.0%) | 13 (5.7%) | 5 (1.3%) | 48 (4.6%) |
| J01DD | Third-generation cephalosporins | 554 (6.6%) | 359 (8.0%) | 153 (7.4%) | 24 (10.5%) | 6 (1.5%) | 4 (0.4%) |
| J01DE | Fourth-generation cephalosporins | 101 (1.2%) | 44 (1.0%) | 53 (2.6%) | 2 (0.9%) | 2 (0.5%) | 0 |
| J01DF | Monobactams | 12 (0.1%) | 3 (0.07%) | 5 (0.3%) | 1 (0.4%) | 0 | 2 (0.2%) |
| J01DH | Carbapenems | 323 (3.8%) | 112 (2.5%) | 193 (9.4%) | 13 (5.7%) | 1 (0.3%) | 1 (0.1%) |
| J01EA | Trimethoprim and derivates | 8 (0.09%) | 2 (0.04%) | 1 (0.05%) | 1 (0.4%) | 4 (1.0%) | 0 |
| J01EB | Short-acting sulfonamides | 1 (0.01%) | 0 | 1 (0.05%) | 0 | 0 | 0 |
| J01EC | Intermediate-acting sulfonamides | 8 (0.09%) | 1 (0.02%) | 4 (0.2%) | 1 (0.4%) | 2 (0.5%) | 0 |
| J01EE | Combinations of sulfonamides and trimethoprim | 265 (3.1%) | 72 (1.6%) | 57 (2.8%) | 2 (0.9%) | 109 (27.5%) | 9 (0.9%) |
| J01FA | Macrolides | 306 (3.6%) | 147 (3.3%) | 21 (1.0%) | 6 (2.6%) | 77 (19.4%) | 1 (0.1%) |
| J01FF | Lincosamides | 237 (2.8%) | 158 (3.5%) | 36 (1.8%) | 4 (1.7%) | 4 (1.0%) | 34 (3.3%) |
| J01GB | Aminoglycosides | 123 (1.5%) | 70 (1.6%) | 40 (1.9%) | 3 (1.%) | 6 (1.5%) | 1 (0.1%) |
| J01MA | Fluoroquinolones | 1131 (13.4%) | 658 (14.6%) | 315 (15.3%) | 37 (16.2%) | 36 (9.1%) | 61 (5.9%) |
| J01MB | Other quinolones | 1 (0.01%) | 0 | 1 (0.05%) | 0 | 0 | 0 |
| J01RA | Combinations of antibacterials | 23 (0.3%) | 15 (0.3%) | 3 (0.1%) | 0 | 3 (0.8%) | 0 |
| J01XA | Glycopeptide antibacterials | 239 (2.8%) | 83 (1.8%) | 145 (7.0%) | 4 (1.7%) | 0 | 3 (0.3%) |
| J01XB | Polymyxins | 15 (0.2%) | 7 (0.2%) | 7 (0.3%) | 0 | 1 (0.3%) | 0 |
| J01XD | Imidazole derivatives | 202 (2.4%) | 112 (2.5%) | 42 (2.0%) | 0 | 2 (0.5%) | 38 (3.7%) |
| J01XE | Nitrofuran derivatives | 131 (1.6%) | 49 (1.1%) | 26 (1.3%) | 4 (1.7%) | 31 (7.8%) | 0 |
| J01XX | Other antibacterials | 51 (0.6%) | 16 (0.4%) | 23 (1.1%) | 0 | 10 (2.5%) | 14 (1.4%) |

ATC= Anatomical Therapeutic Chemical classification system, CAI = community-acquired infections, ECDC = European Centre for Disease Prevention and Control, HAI = healthcare-associated infections, LAI = Infection present on admission from long-term care facility or Nursing Home, MP = medical prophylaxis, SP = surgical prophylaxis
