# Supplementary material for: Point prevalence survey of antimicrobial use and healthcare-associated infections in Belgian acute care hospitals: results of the Global-PPS and ECDC-PPS 2017
Source: Antimicrob Resist Infect Control. 2020 Jan 13;9:13. doi: 10.1186/s13756-019-0663-7 (PMC6958935; doi:10.1186/s13756-019-0663-7)
Supplement: Supplementary file 3 — Additional file 3: Table S3. Description of diagnosis sites of the antimicrobial prescriptions per indication, total results for Global and ECDC-PPS 2017 (Belgium, acute care hospitals). [file 13756_2019_663_MOESM3_ESM.pdf]

Point Prevalence Survey of Antimicrobial Use and Healthcare-Associated Infections in Belgian Acute Care Hospitals: Results of the Global-PPS and ECDC-PPS 2017

ADDITIONAL FILE 3

Table S3: Description of diagnosis sites of the antimicrobial prescriptions per indication, total results for Global and ECDC-PPS 2017 (Belgium, acute care hospitals)

**Diagnosis site**	**Number of prescriptions (% all diagnosis sites)**					
	Total	CAI	HAI	LAI	MP*	SP*
**All diagnosis sites**	**9232**	**4775**	**2333**	**248**	**545**	**1038**
Central Nervous System infections (CNS)	147 (1.6%)	82 (1.7%)	35 (1.5%)	0	4 (0.7%)	26 (2.5%)
Cardiovascular System infections (CVS)	193 (2.1%)	102 (2.1%)	60 (2.8%)	0	1 (0.2%)	27 (2.6%)
Ear, Nose, Throat infections (ENT)	215 (2.3%)	140 (2.9%)	44 (1.9%)	4 (1.6%)	7 (1.3%)	19 (1.8%)
Eye infections (EYE)	7 (0.08%)	6 (0.1%)	0	0	0	1 (0.1%)
Gastro-Intestinal tract infections (GI)	1176 (12.7%)	733 (15.4%)	351 (15.1%)	18 (7.3%)	8 (1.5%)	65 (6.3%)
Obstetric/ Gynaecological infections (GUOB)	109 (1.2%)	65 (1.4%)	19 (0.8%)	0	8 (1.5%)	14 (1.3%)
Respiratory infections (RESP)	2365 (25.6%)	1576 (33.0%)	606 (26.0%)	110 (44.4%)	59 (10.8%)	8 (0.8%)
Skin and soft Tissue + Bone/joint infections (SSTBJ)	1539 (16.7%)	844 (17.7%)	400 (17.2%)	29 (11.7%)	4 (0.7%)	257 (24.8%)
Urinary Tract infections (UTI)	1232 (13.3%)	685 (14.4%)	373 (16.0%)	70 (28.2%)	37 (6.8%)	66 (6.4%)
Neonatal	23 (0.2%)	7 (0.1%)	0	0	15 (2.8%)	0
No defined site	2226 (24.1%)	535 (11.2%)	445 (19.1%)	17 (6.9%)	146 (26.8%)	2 (0.2%)
Missing data					*256 (47.0%)*	*553 (53.3%)*

CAI = community-acquired infections, ECDC = European Centre for Disease Prevention and Control, HAI = healthcare-associated infections, LAI = Infection present on admission from long-term care facility or Nursing Home, MP = medical prophylaxis, SP = surgical prophylaxis

* Diagnosis sites for prophylaxis only registered in the Global-PPS
