# Supplementary material for: Point prevalence survey of antimicrobial use and healthcare-associated infections in Belgian acute care hospitals: results of the Global-PPS and ECDC-PPS 2017
Source: Antimicrob Resist Infect Control. 2020 Jan 13;9:13. doi: 10.1186/s13756-019-0663-7 (PMC6958935; doi:10.1186/s13756-019-0663-7)
Supplement: Supplementary file 4 — Additional file 4: Table S4. Overview of the number of isolates (selected bug-drug combinations) with known antimicrobial susceptibility testing results (AST; first-level antimicrobial resistance (AMR) markers combined) for healthcare-associated infections (HAIs) and resistant results to the antimicrobials included in the protocol, ECDC-PPS 2017 (Belgium, acute care hospitals). [file 13756_2019_663_MOESM4_ESM.pdf]

Point Prevalence Survey of Antimicrobial Use and Healthcare-Associated Infections in Belgian Acute Care Hospitals: Results of the Global-PPS and ECDC-PPS 2017

ADDITIONAL FILE 4

Table S4: Overview of the number of isolates (selected bug-drug combinations) with known antimicrobial susceptibility testing results (AST; first-level antimicrobial resistance (AMR) markers combined) for healthcare-associated infections (HAIs) and resistant results to the antimicrobials included in the protocol, ECDC-PPS 2017 (Belgium, acute care hospitals)

Microorganisms	Number of isolates	Number of isolates with a known AST result	Number of susceptibility tests with a resistant result for the included antimicrobials			
			C3G	CAR	GLY	OXA
Staphylococcus aureus	81	80	-	-		7
Enterococcus faecium	11	11	-	-	1	-
Enterococcus faecalis	44	42	-	-	1	-
Escherichia coli	162	160	29		-	-
Klebsiella pneumonia	38	37	18		-	-
Klebsiella oxytoca	13	13	3	1	-	-
Klebsiella spp., not specified	2	2			-	-
Enterobacter cloacae	38	36	14	2	-	-
Enterobacter aerogenes	10	9	2		-	-
Enterobacter agglomerans	1	1			-	-
Proteus mirabilis	20	18	1		-	-
Proteus vulgaris	3	3	2		-	-
Proteus spp., other	1	1			-	-
Morganella spp.	13	10	4		-	-
Serratia marcescens	13	11	1		-	-
Citrobacter koseri	7	7	1		-	-
Citrobacter freundii	5	5	3	1	-	-
Citrobacter spp., other	1	1	1		-	-
Acinetobacter baumannii	4	4	-		-	-
Pseudomonas aeruginosa	47	44	-	4	-	-

AST = antimicrobial susceptibility testing, C3G = third-generation cephalosporins; CAR = carbapenems; ECDC = European Centre for Disease Prevention and Control, GLY = glycopeptides; OXA = oxacilline; spp. = species; - = not registered
